# Comparative Dosimetry of Single and Hybrid ^177^Lu, ^161^Tb, and ^90^Y in PSMA-Targeted Therapy

**DOI:** 10.3390/diseases14090305

**Published:** 2026-08-24

**Authors:** Olatunde Michael Oni, Tim A. D. Smith

**Affiliations:** 1 Nuclear Futures Institute, School of Computer Sciences and Engineering, Bangor University, Dean Street, Bangor LL57 1UT, UK; ltn24nrt@bangor.ac.uk; 2 Division of Cancer Sciences, University of Manchester, Manchester M20 4GJ, UK

**Keywords:** targeted radionuclide therapy, PSMA PET, voxel-based dosimetry, Monte Carlo, dose-point kernel, ^161^Tb, ^177^Lu, ^90^Y, equivalent uniform dose, tumour control probability

## Abstract

Background: Patient-specific targeted radionuclide therapy (TRT) requires consideration not only of administered activity but also of the spatial distribution of radiopharmaceutical uptake and radionuclide-specific energy deposition. This study developed a voxel-based computational workplan to compare ^177^Lu, ^161^Tb and ^90^Y, together with hybrid radionuclide models, using patient-specific PSMA PET-derived tumour activity distributions. Methods: PSMA PET/CT data from 20 patients with prostate cancer, comprising 10 ^18^F-PSMA and 10 ^68^Ga-PSMA examinations, were processed to obtain 2285 quality-filtered lesions. Radionuclide-specific dose-point kernels (DPKs) were generated in water using OpenGATE and applied to voxel-wise lesion activity distributions to reconstruct absorbed-dose maps. Kernel characteristics were evaluated using radial energy-containment metrics, and ^177^Lu, representing ^161^Tb simulations, was subjected to grid-convergence testing and external comparison with a published DPK. Lesion dosimetry was assessed using Dmean, D90, D95, equivalent uniform dose (EUD) and tumour control probability (TCP), with uncertainty quantified using patient-cluster bootstrap confidence intervals. Kinetic sensitivity and diagnostic tracer subgroup analyses were additionally performed. Results: The study showed that ^161^Tb produced the highest median lesion-level Dmean, D90, D95 and EUD at 182.79, 136.28, 132.07 and 148.53 Gy, respectively, with a median TCP of 0.981. Corresponding values for ^177^Lu were 141.33, 105.42, 102.10 and 114.78 Gy (TCP 0.930), while ^90^Y produced lower local dose metrics but the broadest radial dose distribution, consistent with its longer-range β-particle crossfire. ^161^Tb remained the highest-ranking radionuclide across the investigated kinetic cases and within both diagnostic tracer subgroups. Hybrid ^161^Tb/^90^Y kernels provided a controllable compromise between localised energy deposition and extended crossfire; a 70:30 model increased central dose localisation while retaining an R90 and R95 of 6 and 7 mm, respectively. Grid-convergence and published-DPK comparisons supported the numerical adequacy of the kernel methodology. Radionuclide emission characteristics substantially influence the transformation of heterogeneous tumour uptake into spatial absorbed-dose distributions. Within this model, ^161^Tb provided the strongest overall lesion-level dosimetric performance, whereas the extended range of ^90^Y may offer complementary crossfire for selected bulky or heterogeneous lesions. Conclusions: The findings support phenotype-informed radionuclide comparison and provide a computational basis for investigating hybrid strategies. However, the absolute dose estimates and proposed radionuclide combinations remain model-based and require validation using serial therapeutic imaging, heterogeneous patient-specific dosimetry and normal-organ dose constraints before clinical translation.

## 1. Introduction

Targeted radionuclide therapy (TRT) is an important approach to precision oncology in nuclear medicine, with the potential to use information derived from molecular imaging to characterise radiopharmaceutical uptake and guide therapeutic radiation delivery to disease sites. In routine practice, however, many treatments are still prescribed using fixed or body-weight-adjusted administered activities. Although clinically convenient, this approach does not fully exploit the quantitative information available from diagnostic and post-therapy imaging. Patients receiving the same administered activity may exhibit markedly different tumour uptake, normal-organ clearance and intratumoural dose distributions. Furthermore, lesions within the same patient may differ in size, PSMA expression, uptake heterogeneity and radiosensitivity.

Absorbed dose (Gy) remains the central dosimetric quantity linking radiopharmaceutical localisation to biological effect [1]. In TRT, absorbed dose depends on patient-specific anatomy, time-dependent activity distribution and radionuclide emission characteristics [1,2]. The dose delivered to a region comprises contributions from activity within that region (self-dose) and, for emissions with sufficient range, activity in neighbouring regions (cross-dose or crossfire) [1]. This distinction is particularly important in tumours with heterogeneous radiopharmaceutical uptake, where a mean absorbed dose may obscure spatially underdosed regions.

Voxel-based dosimetry (VBD) addresses this limitation by retaining the three-dimensional spatial distribution of activity measured by PET or SPECT and converting it into a corresponding three-dimensional absorbed-dose distribution. Three principal approaches have been described for this conversion: local energy deposition, dose-point/dose-voxel kernel convolution, and full Monte Carlo radiation transport [1,3]. Full Monte Carlo transport is generally considered the most physically comprehensive because it can explicitly model particle transport, tissue heterogeneity and complex geometries, although at considerable computational cost [2,3]. Kernel-based convolution has notably provided a computationally efficient alternative and can closely approximate Monte Carlo-derived dose distributions in tissue when its underlying assumptions are appropriate [2,4].

Simulated dose kernels have thus provided a practical means of combining detailed radiation-transport modelling with computationally efficient patient-specific voxel dosimetry. Integrating conventional techniques in medical internal radiation dosimetry (MIRD) with computational modalities has strengthened previous studies in establishing radionuclide-specific dose-point and dose-voxel kernels as worthwhile utility for voxel-level absorbed-dose calculations [2,3,4,5,6,7,8,9]. Such kernels are particularly relevant when comparing radionuclides with substantially different emission spectra and particle ranges because the same heterogeneous activity distribution may produce different spatial dose distributions according to the underlying radiation-transport characteristics.

Following similar concepts, this study adopts Monte Carlo simulation to generate radionuclide-specific dose kernels and applies them to patient-specific PSMA PET-derived lesion activity maps. The radionuclides investigated include ^177^Lu, a clinically established radionuclide for PSMA-targeted β-particle therapy; ^161^Tb, which has β-emission characteristics broadly comparable to ^177^Lu but additionally emits conversion and Auger electrons that can enhance short-range energy deposition; and ^90^Y, a higher-energy β-emitter with a longer particle range and therefore greater potential for crossfire across heterogeneous tumour regions. Individual radionuclides and selected ^161^Tb/^90^Y and ^177^Lu/^90^Y combinations were evaluated using the same lesion activity distributions to determine how radionuclide emission characteristics and complementary particle ranges influence spatial absorbed-dose deposition.

The study further integrates lesion-level radiomic characterisation with voxel dosimetry to investigate whether radionuclide-specific dosimetric performance varies according to tumour phenotype and uptake heterogeneity. This investigation establishes and evaluates a patient-specific computational framework for comparing single and combined radionuclide strategies to determine whether complementary emission characteristics could provide a rationale for phenotype-informed optimisation of targeted radionuclide therapy.

## 2. Materials and Methods

### 2.1. Overall Workplan

**Figure 1:**
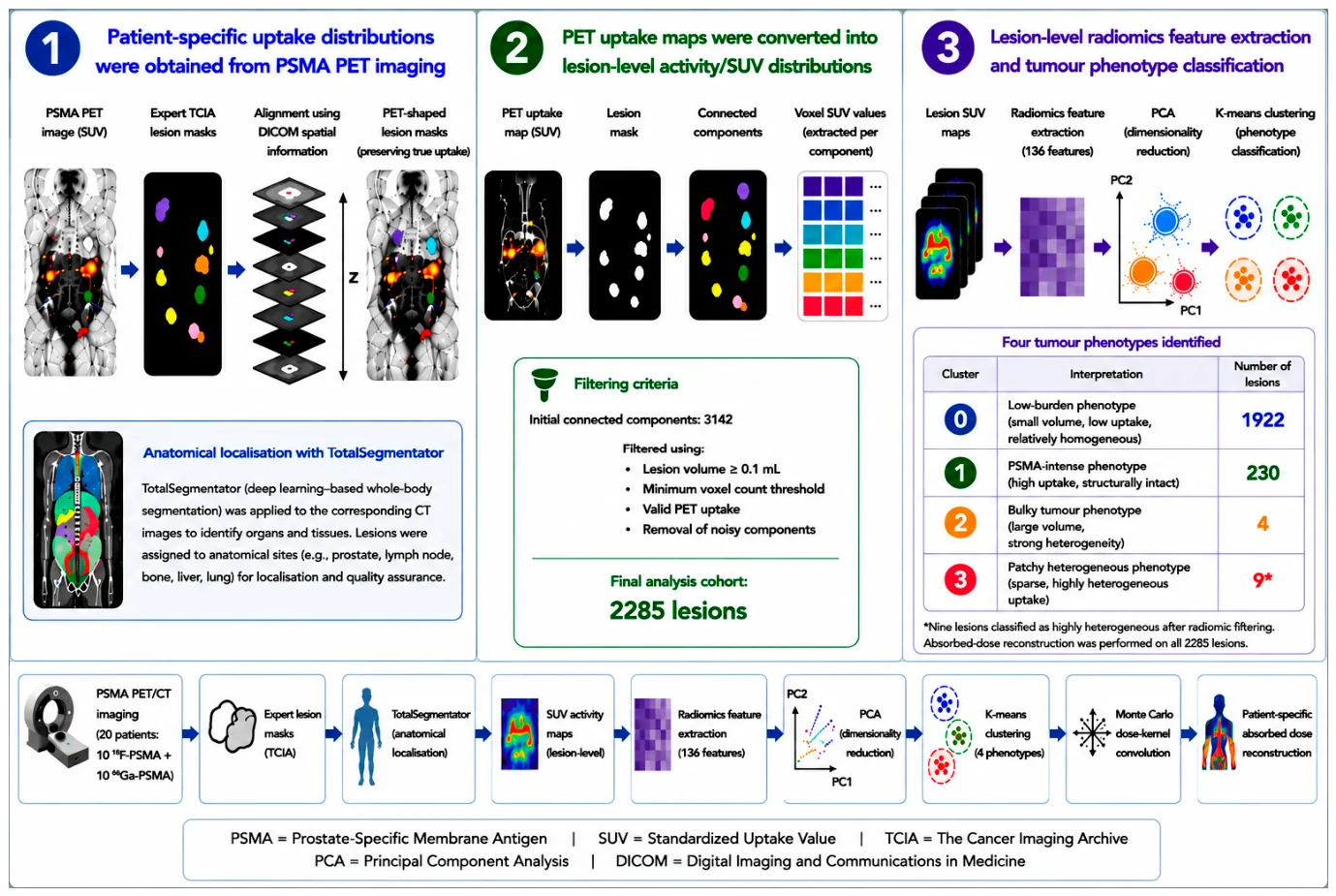
Overview of the computational workplan for patient-specific PSMA PET voxel dosimetry, adopting radiomics feature extraction, tumour phenotype classification, and Monte Carlo absorbed-dose reconstruction (figure created with OpenAI GPT-5.6 Sol).

The patient-specific uptake distribution was acquired from the PSMA PET images accessed from the cancer imaging archive (TCIA). After the pre-processing of the PET/CT imaging dataset in DICOM format, relevant libraries in Python 3.11.6 computer programming package were engaged for simulation and other computational tasks throughout the investigation procedures. In addition to the manual segmentation annotation from the accessed dataset, a validated deep learning algorithm (TotalSegmentator) [10] for whole-body segmentation of human organs and tissues was adopted for the segmentation of the prostate from twenty prostate cancer patients from the TCIA database, leading to the extraction of the masks subsequently used for radiomics analysis and tumour phenotype-based dosimetry. The steps and actions involved in image processing, radiomics extraction and phenotype classification are presented in Figure 1.

The twenty prostate cancer patients in the study cohort comprised ten patients imaged using ^18^F-labelled PSMA tracers, while the other ten were imaged using ^68^Ga-labelled PSMA tracers. After lesion extraction and quality-control filtering, 2285 lesions, comprising 2178 lesions from the ^18^F cohort and 107 lesions from the ^68^Ga cohort, were identified for investigation. The marked difference in lesion numbers between the two radioisotopes reflects the characteristics of the publicly available dataset rather than intentional oversampling.

The segmentation of individual lesions was compared (for validation) with the expert-generated binary lesion masks supplied with the TCIA PSMA PET/CT Lesions dataset. The resulting binary masks were subsequently used to isolate tumour voxels while preserving the original PET voxel intensities for subsequent quantitative analysis. To provide anatomical context, the corresponding CT images were processed further using TotalSegmentator, resulting in anatomical segmentations which were used to identify the organ or tissue compartment containing each lesion. This approach ensures anatomical localisation and quality assurance throughout the radiomics and dosimetric analyses, exclusively performed within the extracted lesion masks.

### 2.2. PET Radiomics Feature Extraction

Voxel-based radiomic descriptors were extracted directly from the PET activity distributions within each lesion mask using Python-based image analysis tools following the recommendations of the Image Biomarker Standardisation Initiative (IBSI). First-order intensity statistics included the mean, maximum and sum standard uptake values—SUV_mean_, SUV_maximum_ and SUV_sum_, respectively.

For each segmented lesion, the total tumour volume $V_{i}$ was calculated from the number of segmented voxels and the corresponding PET voxel dimensions according to

(1) $$V_{i}=N_{i}V_{voxel}$$

where $N_{i}$ represents the number of voxels contained within the lesion mask, and $V_{voxel}$ is the physical volume of a single PET voxel.

To facilitate comparison between the ith lesions of different morphologies, an equivalent spherical radius $R_{i}$ was calculated, assuming volume preservation.

(2) $$R_{i}={\left(\frac{3V_{i}}{4\pi }\right)}^{1/3}$$

Lesions were subsequently categorised according to equivalent diameter into small, intermediate and large tumours to make phenotype-specific dosimetric analyses possible for comparison of the metrics. Grey-Level Co-occurrence Matrix (GLCM), Grey-Level Run Length Matrix (GLRLM), Grey-Level Size Zone Matrix (GLSZM), and Grey-Level Dependence Matrix (GLDM), defined and standardised by the IBSI, were the radiomic features extracted as spatial heterogeneity markers within the tumours. Defining the co-occurrence probability matrix as P(i−j), GLCM contrast and GLCM homogeneity are respectively expressed as

(3) $$\mathrm{Contrast}=\sum_{i,j}{\left(i-j\right)}^{2}P\left(i-j\right)$$

(4) $$\mathrm{Homogeneity}=\sum_{i,j}\frac{P\left(i-j\right)}{1+|i-j|}$$

Other feature extractions considered in this study included the Grey-Level Run Length Matrix (GLRLM), which, in addition to characterising the lengths of contiguous runs of voxels sharing identical intensity values, also provides measures of texture continuity [11]. The Grey-Level Size Zone Matrix (GLSZM) feature, which quantifies the size distribution of homogeneous voxel clusters and is particularly sensitive to tumour heterogeneity and lesion fragmentation [12], was also considered.

### 2.3. First-Order Entropy and Gini Heterogeneity Index

After the extraction of the features, first-order entropy was calculated to quantify randomness in the voxel-wise PET intensity distribution. Following the Image Biomarker Standardisation Initiative (IBSI), entropy for discretised intensity distribution was defined as

(5) $$H=-\sum_{i=1}^{N_{g}}p_{i}{log}_{2}\left(p_{i}\right),$$

where $p_{i}$ is the probability of intensity level i, and $N_{g}$ is the number of discretised intensity levels. Higher entropy indicates greater randomness or heterogeneity of intralesional tracer uptake. The definition and standardisation of first-order radiomic features followed the IBSI framework [13].

Inequality of the voxel-wise PET uptake distribution was additionally quantified using the Gini coefficient, expressed for a lesion containing n voxels with non-negative intensity values $x_{i}$ and mean intensity $\bar{x}$ as follows [14,15]:

(6a) $$G=\;\frac{1}{2n^{2}\bar{x}}\sum_{i=1}^{n}\sum_{j=1}^{n}\left|x_{i}-x_{j}\right|.$$

The normalised mean absolute difference representation of the Gini coefficient, originally introduced by Gini, was translated to a mathematically equivalent formulation [14,15], presented in Equation (6a), and applied in this study; this was obtained by arranging the voxel intensities in ascending order, $x_{\left(1\right)}\leq \ldots \leq x_{\left(n\right)},$ to produce an equivalent rank-based discrete representation of the Gini coefficient as follows:

(6b) $$G=\;\frac{2\sum_{i=1}^{n}i{\;x}_{\left(i\right)}}{n\sum_{i=1}^{n}x_{\left(i\right)}}-\;\frac{n+1}{n}.$$

The value *G* = 0 represents equal voxel intensities, whereas progressively large values indicate an increasing inequality of intralesional tracer uptake.

In this study, Equation (6a) was used because the lesion data consisted of discrete voxel-wise PET intensities.

### 2.4. Feature Normalisation and Dimensionality Reduction and Tumour Phenotype Classification

To minimise scale-dependent bias between radiomic variables, each feature was standardised using z-score normalisation:

(7) $$z_{i}=\frac{x_{i}\;-\;\mu }{\sigma }$$

where $x_{i}$ denotes the original feature value, and μ  and σ are respectively the feature mean and the corresponding standard deviation. Principal Component Analysis (PCA) was performed to reduce feature redundancy while preserving the dominant sources of biological variability. The transformed feature vector was thereafter obtained from

(8) Z=XW

where X is the normalised feature matrix, W contains the eigen vectors of the covariance matrix, and Z represents the principal component scores.

Unsupervised K-means clustering with the reduced PCA, expressed in Equation (9), was adopted to classify lesions:

(9) $$J=\;\sum_{k=1}^{K}\sum_{x_{i}\in C_{k}}|\left|x_{i}-\mu_{k}\right|{|}^{2}$$

Defining K as the number of clusters, $C_{k}$ as a representation of cluster k, and $\mu_{k}$ as the centroid of cluster k, the optimal solution identified four biologically distinct tumour phenotypes, namely the following:

Cluster 0: Low-burden lesions, consisting of small tumour volume, and relatively homogenous.

Cluster 1: PSMA-intense lesions, exhibiting high radiotracer uptake with preserved structural integrity.

Cluster 2: Bulky tumours characterised by large lesion volume and substantial intratumoural heterogeneity.

Cluster 3: Patchy heterogenous lesions demonstrating sparse and highly heterogeneous activity distributions with fragmented uptake patterns.

These phenotype classes were subsequently propagated throughout the absorbed-dose reconstruction, enabling a direct comparison of radionuclide performance across the different tumour phenotypes.

Statistical Considerations for Lesion-Level Analyses

Dosimetric metrics were evaluated at the lesion level while considering the structure of the dataset. The 2285 lesions from 20 patients, with each lesion evaluated under the five radionuclide treatment models (^177^Lu, ^161^Tb, ^90^Y, ^177^Lu/^90^Y and ^161^Tb/^90^Y), generated 11,425 lesion-model dose reconstructions. Consequently, neither lesions originating from the same patient nor the five treatment models for an individual lesion were considered statistically independent. Due to the distributions of lesion-level absorbed-dose and radiobiological endpoints being non-Gaussian and, in the case of TCP, bounded, Dmean, D90, D95, EUD and TCP were summarised using the median and interquartile range (IQR). Uncertainty in cohort-level median estimates was additionally quantified using 95% confidence intervals obtained by cluster bootstrap resampling at the patient level, thereby retaining all lesions and corresponding observations of the treatment model belonging to each sampled patient.

Overall differences among radionuclide treatment models were assessed using repeated-measures comparisons in which treatment model constituted the within-lesion factor. Pairwise comparisons between treatment models were performed following the corresponding overall comparison. To control the family-wise error arising from multiple pairwise comparisons, adjusted *p*-values were calculated using the Holm procedure, leading to the determination of statistical significance at the 0.05 level. Effect estimates and their 95% confidence intervals were reported alongside adjusted *p*-values, with emphasis placed on effect magnitude and consistency rather than statistical significance alone.

The clustering nature of lesions within patients necessitated patient-level sensitivity analyses, which were additionally performed by calculating the median endpoint for each treatment model within each patient before between-model comparison. This provided 20 independent patient-level observational units and was used to determine whether the principal radionuclide ranking observed in the lesion-level analysis remained evident without allowing patients with large numbers of lesions to dominate the inference.

### 2.5. Monte Carlo Transport and Dose-Point Kernel Generation

Dose-point kernels (DPKs) were generated using OpenGATE version 10.0.2, executed under Python 3.11.5. The OpenGATE toolkit operated the generic Geant4 (version 11.3) with multithreading capability, employing geometry consisting of an isotropic point source positioned at the centre of a homogeneous G4_WATER phantom. The production kernels used a three-dimensional Cartesian scoring matrix with an isotropic voxel dimension of 1 mm x 1 mm x 1 mm chosen for spatial binning of the deposited energy. The energy deposition and absorbed dose were recorded using the OpenGATE DoseActor after instantiating the radionuclides as radioactive ions, with radioactive decay process enabled through the Geant4 decay physics constructors. Electromagnetic interactions were ensured in the simulation procedure by adopting EmStandardPhysics_option4. Particularly relevant to ^161^Tb, due to the contribution of its conversion and Auger electrons to highly localised energy deposition, atomic de-excitation processes, including fluorescence, Auger electron production, Auger cascades and particle-induced X-ray emission (PIXE) were enabled.

Within the water phantom, Geant4 production range cuts of 0.01 mm (10 µm) were specified independently for photons, electrons and positrons, while a 10 mm production cut was applied to the surrounding world volume. The 1 mm voxel resolution employed in this study was from previous work, with voxel sizes ranging from 0.1 mm to 10 cm [3,4] for similar investigations, particularly for ^177^Lu, which bears characteristics close to ^161^Tb. The resolution choice was additional traceable to the 1 mm voxel spacing presented in the MIRD Pamphlet No. 17 [8], which established the voxel-S-value framework for non-uniform internal dosimetry. The report specifically notes that PET/SPECT activity distributions are represented by voxels in the order of several millimetres, with tabulated clinical voxel S values presented at 3 and 6 mm dimensions.

The DPK outputs were stored as MetaImage files and analysed using radial profiles and cumulative dose fraction curves translating to R50, R90 and R95, denoting the radius containing 50%, 90% and 95% of the total deposited dose within the kernel grid.

#### 2.5.1. DPK Grid Convergence and External Validation for ^177^Lu

The influence of scoring-grid resolution on the ^177^Lu dose-point kernel (as a representative of ^161^Tb) was evaluated using isotropic voxel sizes of 0.25, 0.5, 1.0 and 2.0 mm in water, with a total of 10^6^ radioactive decays for each resolution. The 0.25 mm calculation was treated as the highest resolution and selected for numerical reference. Convergence was assessed using total deposited energy per decay, cumulative deposited energy fractions within fixed physical radii (1, 2 and 5 mm), source-containing voxel energy fractions and radial energy-containment metrics (R50, R90 and R95). An independent radial-shape benchmark was additionally performed using the published ^177^Lu water DPK dataset of Papadimitroulas et al. [16]. The 0.25 mm three-dimensional OpenGATE kernel was converted to a radial representation and interpolated to the radial coordinates of the published datasets to determine the pointwise symmetric difference, calculated using

(10) $$Difference\;\left(\%\right)=\frac{\left|D_{OpenGATE}-D_{Published}\right|}{max(D_{OpenGATE},\;D_{Published})}\times 100$$

where $D_{OpenGATE}$ and $D_{Published}$ respectively denote dose values from the OpenGATE simulation (this study) and the Published Dose.

Comparisons were performed over 0.5–40 mm and, separately, over 2–40 mm to assess the influence of the immediate source-containing region. Quantification of the agreement was done with mean absolute percentage difference (MAPE), symmetric percentage difference and cumulative radial energy distribution.

#### 2.5.2. Generation of Hybrid Dose-Point Kernels

Following similar a concept of Tandem radionuclide therapy, which presented ^177^Lu/^90^Y with different β-particle ranges combined to exploit the complementary irradiation of small and large lesions [17,18] using a combination of complementary radionuclides, microscopic and macroscopic dose deposition from dose-point kernels was undertaken in this study under the assumption of independent radioactive decays and hybrid kernels generated by weighted linear superposition of the parent kernels. Defining $K_{i}(r)$ and $K_{j}(r)$ as the three-dimensional dose kernels for the complimentary radioisotopes i and j, with r and w respectively representing the spatial position relative to the source voxel and the fractional contribution of the complimentary radioisotopes, the hybrid kernel was calculated as

(11) $$K_{hybrid}\left(r\right)=w_{i}K_{i}\left(r\right)+w_{j}K_{j}(r)$$

Equal weighting (50:50) was initially set to provide a balanced combination of the short-range and long-range beta emissions. Systematic variation in the weighting factor was subsequently performed to identify combinations capable of increasing microscopic dose while preserving the clinically important crossfire contribution. Different cases of combination therapy to optimise the hybrid were subsequently evaluated by varying the relative activities of the combining radionuclides and comparing tumour-level absorbed-dose and radiobiological endpoints. For example, the combination involving ^161^Tb and ^90^Y in the ratio 70:30 corresponded to 3.7 GBq ^161^Tb and 1.85 GBq ^90^Y (66.7:33.3% of total administered activity). The optimisation performed in this study was, however, based on tumour dosimetry, representing a tumour dose rather than a clinically established activity prescription.

### 2.6. Patient-Specific Dose Reconstruction and Radiobiological Endpoints

The diagnostic PET SUV distribution was used to construct the patient-specific dose from the SUV-derived therapeutic activity distribution, which was subsequently time-integrated under specified pharmacokinetic assumptions. From the understanding of voxel-level body-weight-normalised SUV, the initial activity concentration of each voxel was determined by

(12) $$C_{i\;}\left(t_{0}\right)={SUV}_{i}\frac{A_{admin}}{W}$$

where $C_{i\;}\left(t_{0}\right)$ denotes the initial activity concentration (Bq/mL), $A_{admin}$ is the administered activity specified for the therapeutic case (Bq), and W is patient body mass (g). This translates to an initial voxel activity

(13) $$A_{i}\left(t_{0}\right)=C_{i}(t_{0})V_{vox}$$

where $V_{vox}$ is the PET voxel volume (mL). With the assumption of mono-exponential effective clearance, the voxel-level TIA in Bq.s is given as

(14) $${\tilde{A}}_{i}=A_{i}(t_{0})\frac{T_{\frac{1}{2}\;,\;eff}}{\ln2}$$

This subsequently leads to the voxel-wise TIA distribution, expressed as

(15) $${\tilde{A}}_{i}={SUV}_{i}\frac{A_{admin}}{W}\;V_{vox}\frac{T_{\frac{1}{2}\;,\;eff}}{\ln2}$$

However, the absence of therapeutic administered activity data resulted in the use of the diagnostic SUV distribution as a surrogate spatial uptake pattern. A model-based TIA was therefore adopted rather than directly measured. The activity model expressed by Equation (16)

(16) $$A_{i}^{model}\left(0\right)={SUV}_{i}\frac{A_{therapy}}{W}V_{i}$$

further translates to

(17) $${\tilde{A}}_{i}^{model}=A_{i}^{model}\left(0\right)\frac{T_{\;eff}}{\ln2}\;$$

Combining Equations (16) and (17) produces the TIA model:

(18) $${\tilde{A}}_{i}^{model}=\;{SUV}_{i}\frac{A_{therapy}}{W}V_{i}\frac{T_{\;eff}}{\ln2}$$

A three-dimensional absorbed-dose map for each of the radionuclides chosen followed the classical kernel-based dosimetry principle, where a time-integrated activity distribution is convolved with a kernel representing the dose contribution from a unit source voxel [11]. In addition to the mean and maximum dose absorbed (D_mean_ and D_max_) by the tumour, the minimum absorbed dose received by various selected percentages of the lesion volume was a radiobiological metric considered in this study.

The spatial heterogeneity of the reconstructed absorbed-dose distribution within each lesion was quantified using the coefficient of variation (CoV, defined as

(19) $$CoV=\frac{\sigma_{D}}{\bar{D}}$$

where $\sigma_{D}$ is the standard deviation of the voxel absorbed dose, and $\bar{D}$ is the mean absorbed dose.

This was translated to the estimation of tumour control probability (TCP) by calculating the equivalent uniform dose (EUD) following Niemeirko’s formula (Equation (20)) based on the dose required for 50% tumour control. With $D_{i}$ as the absorbed dose in the voxel i, N as the total number of tumour voxels, and a as the tissue-specific volume effect parameter, the EUD was calculated from the reconstructed voxel dose distribution using

(20) $$EUD={\left(\frac{1}{N}\sum_{i=1}^{N}D_{i}^{a}\right)}^{1/a}$$

The EUD-dependent tumour control probability (TCP) was estimated using a logistic model, expressed as

(21) $$TCP=\frac{1}{1+exp[-\gamma \frac{\left(EUD-D_{50}\right)}{D_{50}}]}$$

with commonly adopted values of $D_{50}$ = 50 Gy and γ=2 from radiobiological modelling of prostate tumours [19]. The various metrics were chosen to adequately account for the possible inaccuracy inherent with mean absorbed doses in heterogeneous tumours.

### 2.7. Kinetic Sensitivity Analysis

To quantify the dependence of the reconstructed absorbed dose on the assumed clearance kinetics, a sensitivity analysis was performed for the short-, intermediate- and long-retention conditions of the three radionuclides, with corresponding biological half-lives of 72, 106 and 168 h, respectively, for ^177^Lu, ^161^Tb and ^90^Y. For each radionuclide and condition, the effective half-life was calculated from

$$\frac{1}{T_{\frac{1}{2},eff}}=\frac{1}{T_{\frac{1}{2},\;phys}}+\frac{1}{T_{\frac{1}{2},\;bio}}$$

Using physical half-lives of 6.647, 6.953 and 2.668 days for ^177^Lu, ^161^Tb and ^90^Y resulted in an effective half-life range from 2.067 to 3.409 days for ^177^Lu, while 2.096 to 3.488 days and 1.412 to 1.932 days were recorded for ^161^Tb and ^90^Y. The linear dependence of TIA and, consequently, the absorbed dose with an effective half-life under the assumed mono-exponential model were evaluated relative to the original model assumptions of 3.5 days for ^177^Lu and ^161^Tb and 2.0 days for ^90^Y. Dose volume metrics and EUD were recalculated for each condition, while TCP was recomputed from the case-specific EUD based on its nonlinear dose–response relationship.

### 2.8. Diagnostic Tracer Subgroup Analysis

The potential influence of diagnostic tracer type on SUV-derived dosimetry was evaluated for the ^18^F- and ^68^Ga-labelled PSMA-PET examinations used to construct the modelled therapeutic activity maps. Lesions were stratified according to diagnostic PET tracer and baseline uptake. Lesion characteristics were compared using SUVmean, SUVmax, lesion volume and lesion voxel count. Because multiple lesions originated from individual patients, a patient-level sensitivity analysis was additionally performed to avoid treating lesions from the same patient as statistically independent observations. For each patient, median lesion SUV, lesion volume and dosimetric endpoints (Dmean, D90 and EUD) were calculated, and the resulting independent patient-level distributions were compared between the ^18^F and ^68^Ga groups using the Mann–Whitney U test. The therapeutic radionuclide ranking was additionally evaluated independently within each tracer subgroup to determine whether the principal comparative dosimetric findings were preserved irrespective of diagnostic tracer type.

As a further lesion-level analysis, to take care of the significant lesion number difference between the two tracers, log-transformed dose endpoints were modelled using mixed-effects regression with diagnostic tracer and log-transformed lesion volume as fixed effects.

## 3. Results and Discussion

### 3.1. Image Processing and Lesion Extraction

**Figure 2:**
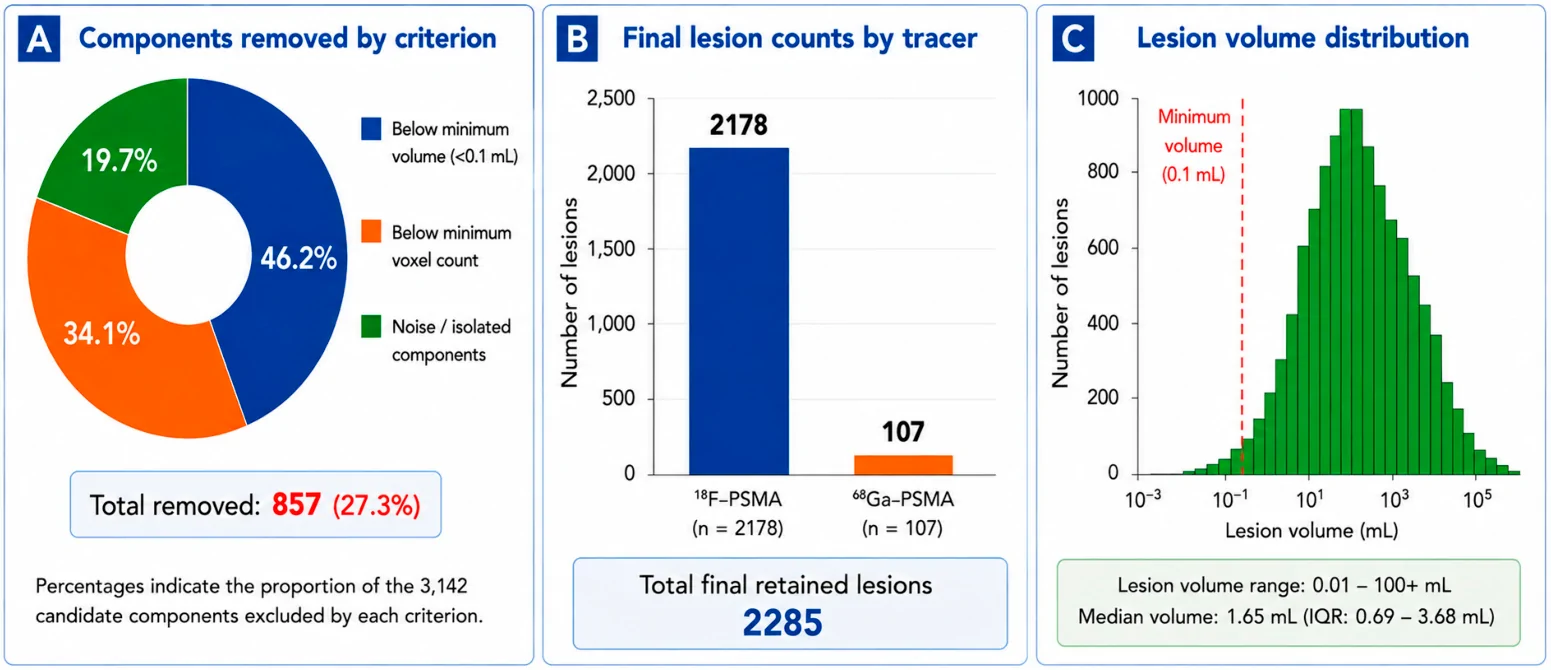
Lesion filtering and final PSMA PET cohort. (**A**) Components excluded by filtering criteria. (**B**) Final lesion counts by ^18^F and ^68^Ga-PSMA tracer. (**C**) Lesion volume distribution, with the dashed line indicating the 0.1 mL inclusion threshold; median volume was 1.45 mL (IQR: 0.49–3.88 mL).

The adoption of initial connected-component analysis produced 3142 candidate tumour components. However, the application of predefined filtering criteria, including minimum lesion volume (≥0.1 mL), minimum voxel counts and the removal of noisy components, identified 2285 biologically meaningful lesions that were retained for subsequent radiomic and dosimetric analyses (Figure 2). The image processing activities of the contributing 2285 lesions after quality filtering, with 95.3% originating from the ^18^F cohort and 4.7% from the ^68^Ga cohort, successfully transformed the patient-specific PSMA PET/CT examinations into quantitative lesion-level activity distributions suitable for radiomic and dosimetric analyses following the workplan (Figure 1).

Subsequent application of the lesion quality filters resulted in substantially more lesions from the ^18^F-PSMA cohort being retained than from the ^68^Ga-PSMA cohort (2178 vs.107 lesions). This difference is likely attributable to the shorter positron range of ^18^F and consequent superior spatial resolution, which improves lesion contrast and reduces partial-volume effects compared with ^68^Ga. The longer positron range of ^68^Ga increases image blurring, particularly in small lesions, making them more susceptible to exclusion by minimum-volume and minimum-voxel filtering criteria. Furthermore, the higher lesion-to-background contrast of ^18^F-PSMA PET facilitates more reliable lesion segmentation and radiomic feature extraction. Consequently, a greater proportion of biologically meaningful lesions were retained for quantitative analysis in the ^18^F cohort. The large number of analysed lesions reflects the comprehensive lesion annotations provided within the TCIA PSMA PET/CT lesions dataset rather than independent patient sampling. Because several lesions originated from the same patient, lesion measurements inevitably exhibited within-patient correlation.

### 3.2. Monte Carlo Dose-Point Kernel Characterisation

#### 3.2.1. Three-Dimensional Dose-Point Kernels

**Figure 3:**
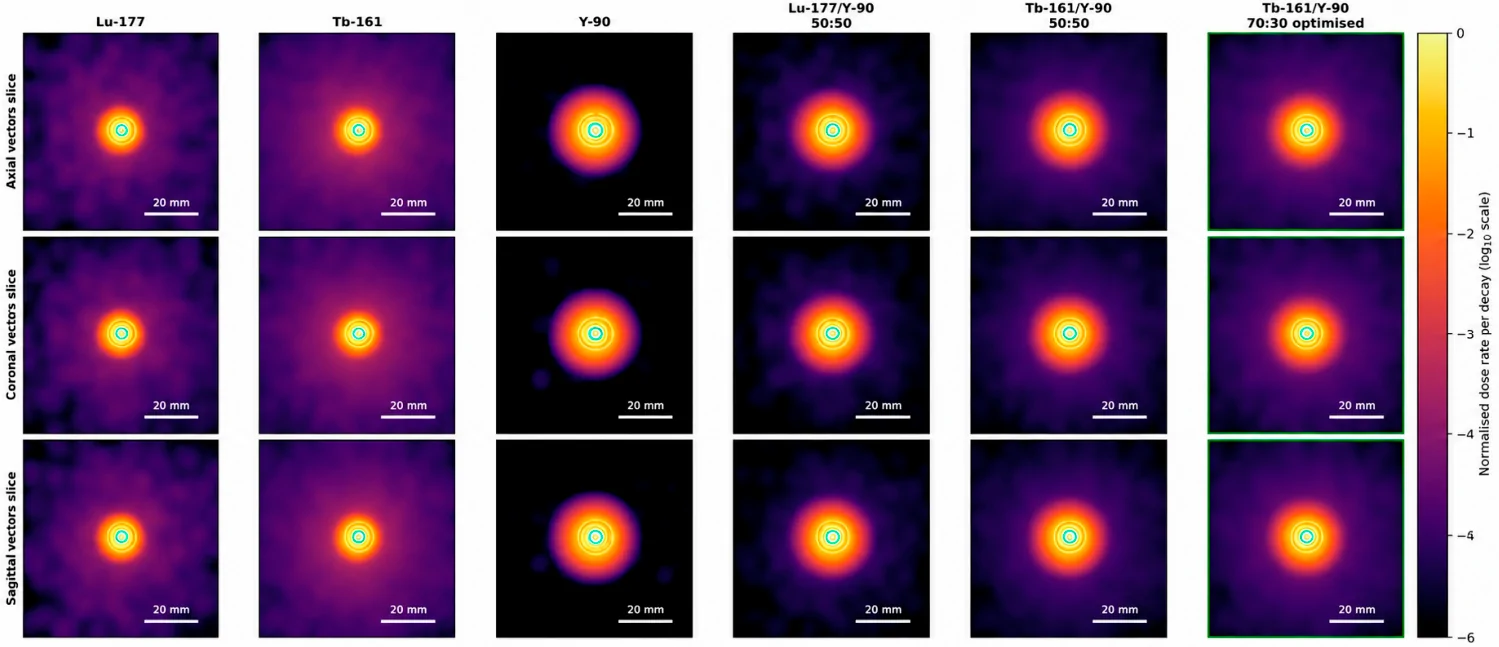
Spatial absorbed-dose distributions across radionuclide models. Axial, coronal and sagittal slices compare ^177^Lu, ^161^Tb, ^90^Y and hybrid models. Cyan contours indicate the lesion region; green outlines identify the optimised ^161^Tb/^90^Y (70:30) model.

The three-dimensional dose-point kernels (DPKs) for ^177^Lu, ^161^Tb, ^90^Y and their hybrid combinations (Figure 3) in a homogeneous water phantom, together with the corresponding radial dose profiles and cumulative dose distributions, revealed distinct differences in energy deposition patterns between the radionuclides. The ^177^Lu kernel exhibited the greatest localisation of deposited energy, with most of the absorbed dose confined to voxels immediately surrounding the decay site. The resulting kernel displayed a compact high-dose core with rapid attenuation away from the source, consistent with the relatively low-energy β-particles emitted by ^177^Lu. The ^161^Tb kernel demonstrated a similarly localised central dose distribution but exhibited a broader low-dose containment than ^177^Lu. This extension reflects the additional contribution of conversion and Auger electrons emitted during ^161^Tb decay, thereby increasing energy deposition over short distances while maintaining excellent localisation around the source voxel.

In contrast, the ^90^Y kernel produced a substantially broader spatial distribution of the absorbed dose. Owing to its high-energy β-emissions, although the majority of the absorbed energy remained concentrated within the first few millimetres from the decay site, ^90^Y demonstrated the broadest radial dose distribution of all radionuclides, producing substantially greater crossfire than ^177^Lu or ^161^Tb.

The hybrid kernels, which displayed intermediate characteristics between their constituent radionuclides, are shown in Figure 3. The 50:50 ^177^Lu/^90^Y combination demonstrated a balanced distribution in which the highly localised component of ^177^Lu was complemented by the longer-range crossfire contribution of ^90^Y. Similarly, the ^161^Tb/^90^Y (50:50) hybrid preserved the strong microscopic dose enhancement associated with ^161^Tb while extending dose coverage through the incorporation of the higher-energy β-particles from ^90^Y.

The Monte Carlo-derived dose-point kernels demonstrated excellent agreement with previously published radionuclide transport characteristics and dose kernel investigations [1,2,3,4]. For ^177^Lu, the simulated R90 value of 2 mm and highly localised energy deposition are consistent with published reports describing the short β-particle range of this radionuclide and its strong confinement of absorbed dose to the immediate vicinity of the decay site [3,4]. The simulated ^161^Tb kernel exhibited similarly high dose localisation but with a broader low-dose envelope than ^177^Lu, reflecting the additional contribution of conversion and Auger electrons that has been widely reported in experimental and computational dosimetry studies [20,21,22,23]. The broadest radial dose distribution was produced by ^90^Y, with R90 and R95 values of 6 mm and 7 mm, respectively, together with the lowest central voxel dose fraction. These observations agree with the well-established higher β-particle energy and longer penetration range of ^90^Y in soft tissue, which produces substantially greater crossfire than lower-energy radionuclides [3,6,24,25].

The hybrid kernels displayed the expected intermediate dosimetric behaviour between their constituent radionuclides. Increasing the proportion of ^161^Tb progressively enhanced microscopic dose localisation, whereas increasing the contribution of ^90^Y extended dose deposition into surrounding tissue through greater crossfire. The optimised ^161^Tb/^90^Y (70:30) kernel increased the central voxel dose fraction from 27.1% for the corresponding 50:50 hybrid to 38.5%, while preserving identical R90 (6 mm) and R95 (7 mm) values. This demonstrates that greater microscopic dose localisation can be achieved without sacrificing the clinically relevant dose coverage provided by the longer-range β-particles of ^90^Y. To the best of our knowledge, this is the first patient-specific study to integrate Monte Carlo-derived hybrid dose-point kernels with PSMA PET-derived tumour phenotypes for the optimisation of radionuclide selection across a large lesion cohort.

#### 3.2.2. DPK Grid Convergence and External Validation

**Figure 4:**
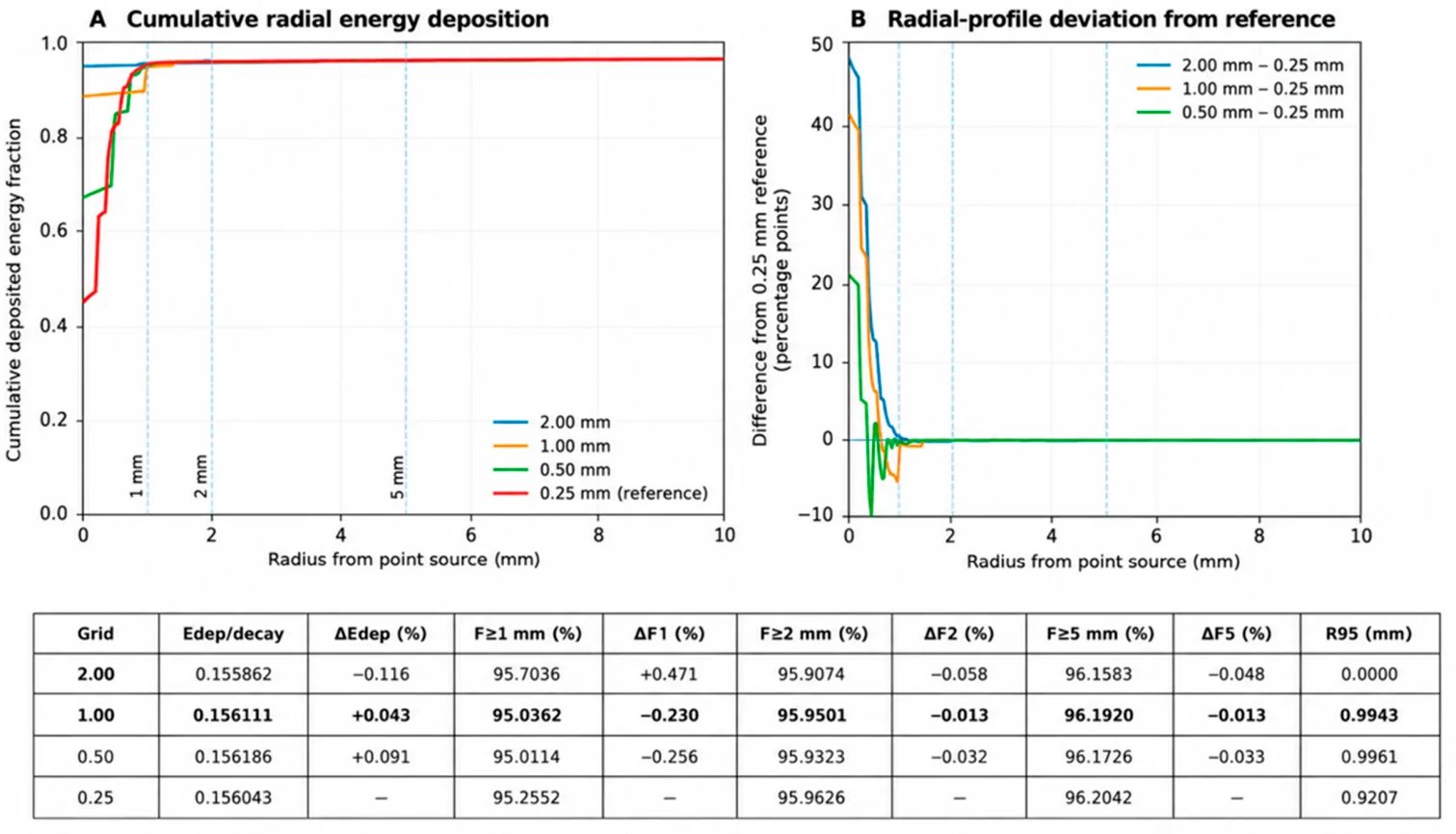
^177^Lu DPK grid-convergence analysis showing (**A**) cumulative radial energy profiles across scoring resolutions and (**B**) percentage deviations relative to the 0.25 mm reference grid. The table summarises total deposited energy, fixed-radius cumulative-energy fractions and R95.

Following the importance of spatial resolution in voxel-based radionuclide dosimetry [26,27], particularly for short-ranged isotopes like ^177^Lu and ^161^TB, a grid-convergence analysis, performed to evaluate the numerical adequacy of the 1 mm voxel resolution for the ^177^Lu DPK using isotropic scoring grids of 0.25, 0.5, 1.0 and 2.0 mm, showed the strong stability of the integral energy deposition quantities with grid refinement (Figure 4). Total deposited energy per decay was respectively 0.156043, 0.156186, 0.156111 and 0.155862 for the 0.25, 0.5, 1.0 and 2.0 mm grids. Relative to the 0.25 mm reference, the 1.0 mm grid differed by only +0.043% in total deposited energy per decay. Differences in cumulative deposited energy fraction for the 1.0 mm grid relative to the 0.25 mm calculation were approximately −0.230%, −0.013% and −0.013% within radii of 1, 2 and 5 mm, respectively. In contrast, near-source containment metrics were more sensitive to voxelisation: R90 was observed to increase from 0.202 mm at 1.0 mm spacing to 0.600 mm at 0.25 mm spacing, whereas R95 changed from 0.994 to 0.921 mm. The source-containing voxel energy fraction decreased progressively from 0.957 at 2.0 mm to 0.470 at 0.25 mm. These findings indicate that grid refinement primarily redistributed energy within the immediate source region while leaving integral and fixed-radius energy deposition quantities essentially unchanged.

**Figure 5:**
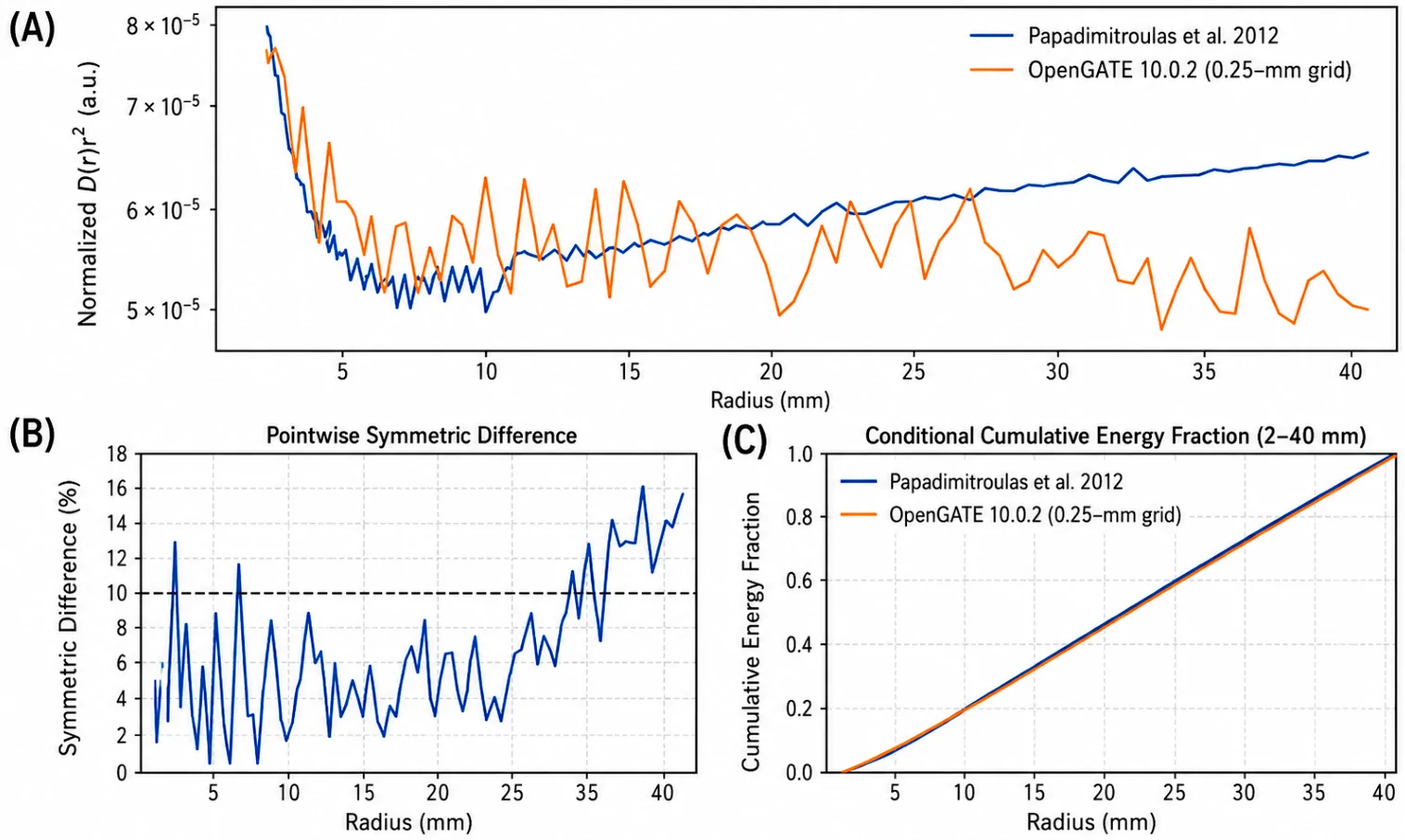
External ^177^Lu DPK validation showing (**A**) radial kernel profiles, (**B**) pointwise symmetric differences, and (**C**) cumulative energy distributions compared with Papadimitroulas et al. [16]. The dashed horizontal line in Panel B represents the benchmark of 10% symmetric difference between the OpenGATE DPK (this study) and the published (Papadimitroulas et al. [16]) DPK.

External comparison of the 0.25 mm ^177^Lu water DPK with the published Monte Carlo kernel of Papadimitroulas et al. [16] demonstrated a close agreement in radial kernel shape (Figure 5). Across 0.5–40 mm, the MAPE and symmetric mean difference were 11.60% and 10.09%, respectively, with $R_{log}^{2}=0.995$. The discrepancy was predominantly localised to the immediate near-source region. Excluding the first 2 mm reduced the MAPE to 4.80%, 4.75% and 5.67% over 2–5, 2–10 and 2–40 mm, respectively, with corresponding symmetric mean differences of 4.51%, 4.46% and 5.49%. The median absolute percentage difference over 2–40 mm was 4.98%, and the cumulative radial energy profiles showed close agreement. Together with the grid-convergence results, these findings support use of the 1.0 mm DPK for subsequent voxel–kernel absorbed-dose reconstruction.

#### 3.2.3. Radial Dose Profiles

**Figure 6:**
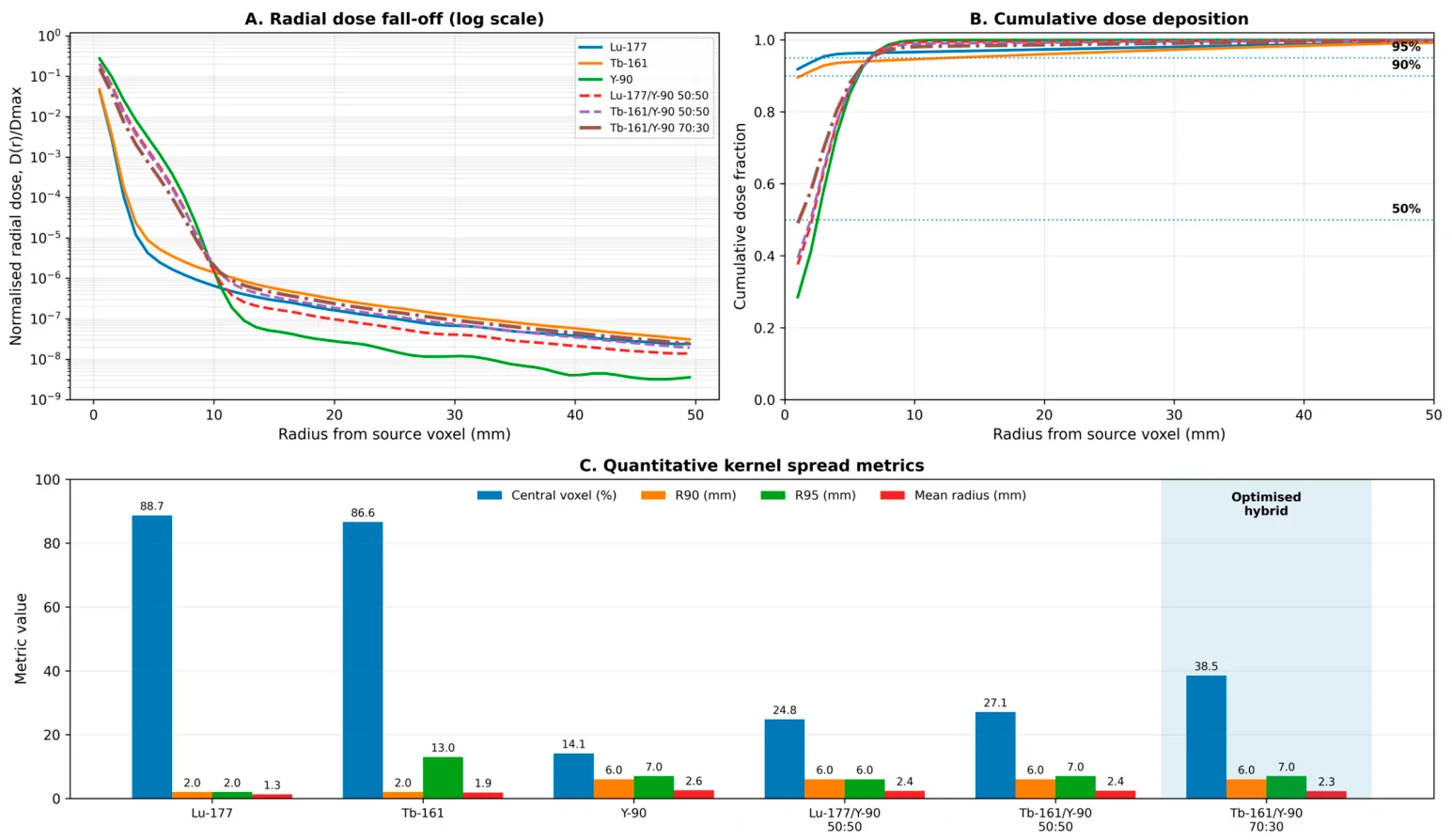
Radial characterisation of Monte Carlo-generated dose-point kernels. (**A**) Normalised radial dose fall-off, (**B**) cumulative dose deposition, and (**C**) central voxel fraction and radial spread metrics (R90, R95 and mean radius) for single and hybrid radionuclide models.

The radial dose profiles demonstrated marked differences in dose fall-off among the simulated radionuclides (Figure 6A). Both ^177^Lu and ^161^Tb exhibited steep exponential reductions in absorbed dose with increasing radial distance from the source, confirming that most emitted energy was deposited within a relatively short range. Among the individual radionuclides, ^161^Tb maintained the highest dose within the first few millimetres owing to its combined β-particle and conversion/Auger electron emissions. The additional low-energy electrons produced a wider local dose envelope than ^177^Lu without substantially increasing long-range dose spread.

Conversely, ^90^Y exhibited the slowest dose attenuation with distance. Its radial profile extended beyond those of all other radionuclides, illustrating its superior ability in the extended irradiation of tissue beyond the immediate vicinity of the decay site through crossfire. The hybrid kernels produced intermediate radial profiles between those of the parent radionuclides. Increasing the proportion of ^161^Tb was found to result in progressively greater dose localisation, whereas increasing the contribution of ^90^Y tended to produce enhanced dose penetration into the surrounding tissue.

#### 3.2.4. Cumulative Dose Deposition

Cumulative dose analysis further highlighted the differences in energy deposition behaviour (Figure 6B). The cumulative dose curves showed that nearly all energy emitted by ^177^Lu was deposited within a very short radial distance, confirming its suitability for treating small, well-defined lesions. Although similarly localised, ^161^Tb presented a slightly larger radius to accumulate the same fraction of deposited energy because of the contribution of conversion and Auger electrons extending beyond the immediate source voxel.

The cumulative dose distribution of ^90^Y increased much more gradually, indicating substantial energy deposition at larger distances. This behaviour is advantageous for irradiating heterogeneous or bulky tumours where complete radionuclide uptake may not occur uniformly throughout the lesion. Hybrid kernels were observed to present a continuous transition between highly localised and broadly distributed dose deposition, thus demonstrating cumulative dose distributions lying between those of the corresponding parent radionuclides.

#### 3.2.5. Optimisation of Hybrid Dose Kernels

The systematic combination of the hybrid kernels, performed by progressively varying the weighting coefficients used in the combination of the DPKs, beginning with an equal 50:50 contribution, identified the most favourable balance between microscopic dose enhancement and macroscopic tumour coverage (Figure 6C). The optimised 70:30 ^161^Tb:^90^Y combination was observed as having an increased central voxel dose fraction from 27.1% to 38.5% while preserving identical R90 (6 mm) and R95 (7 mm) values. These findings indicate that microscopic dose deposition was enhanced without compromising the clinically relevant crossfire range responsible for the deposition of approximately 90–95% of the absorbed energy.

Although the optimised ^161^Tb:^90^Y combination showed favourable tumour dosimetry, clinical implementation of this computational finding would require normal-organ-constrained treatment planning. However, no established clinical evidence have been obtained that ^161^Tb and ^90^Y can be safely co-administered at the specific activity ratio investigated in this study. Emerging ^161^Tb clinical studies have indicated radioisotope activity administration at a GBq scale. A reported activity value of 7.4 GBq was reported [28] as the recommended phase-2 activity in the VIOLET (^161^Tb-PSMA-I&T) study. This value cannot, however, be extrapolated directly to a ^161^Tb/^90^Y combination [29,30]. The maximum deliverable activity of either component was noted to depend on individualised health status, according to kidney, marrow and other organs at risk. As the normal-organ dose contributions from both radionuclides are additive, the present combination is interpreted as a simulation-derived ratio requiring prospective organ-dosimetry and safety validation.

### 3.3. Patient-Specific Absorbed Dose and Tumour Control Probability

The patient-specific absorbed-dose dataset comprised values from 11,425 lesion-model dose records, corresponding to 2285 unique lesions analysed under five treatment models: Lu-177, Tb-161, Y-90, Lu-177/Y-90 and Tb-161/Y-90. As indicated in Figure 2, lesions were derived predominantly from the ^18^F-PSMA group (2178 lesions), with a smaller ^68^Ga-PSMA subset (107 lesions). Size stratification thus identified 1702 small, 539 intermediate and 44 large lesions.

Across the 2285 lesions, ^161^Tb produced the highest median absorbed-dose and radiobiological indices, with Dmean, D90, D95 and EUD of 182.79, 136.28, 132.07 and 148.53 Gy, respectively, and a median TCP of 0.981. The corresponding median values for ^177^Lu were 141.33, 105.42, 102.10 and 114.78 Gy, with a TCP of 0.930, whereas ^90^Y produced the lowest median values (Dmean 65.85 Gy, D90 49.99 Gy, D95 47.53 Gy, EUD 52.99 Gy and TCP 0.530). The observations indicate that, within the treatment models, Tb-161 gave the strongest overall lesion-level dosimetric performance based on the biological feasibility of ^161^Tb’s combination of beta emission with conversion and Auger electron contributions, which have been reported to enhance microscopic energy deposition [22].

The median values, IQRs and patient-cluster bootstrap 95% confidence intervals are reported in Table 1. Overall patient-level repeated-measures comparisons demonstrated significant differences among the five treatment models for Dmean, D90, D95, EUD and TCP (Friedman tests, all *p* < 10^−11^). Pairwise comparisons with Holm correction showed that ^161^Tb remained significantly different from each of the other treatment models for all the metrics. In contrast, no significant differences were detected between ^177^Lu and ^161^Tb/^90^Y for Dmean, D90, D95, EUD and TCP after multiplicity adjustment. Holm-adjusted *p*-values were 0.927 for Dmean, 0.277 for D90, 0.312 for D95, 0.596 for EUD and 0.841 for TCP. Most of the remaining pairwise treatment comparisons were significant after Holm correction.

**Figure 7:**
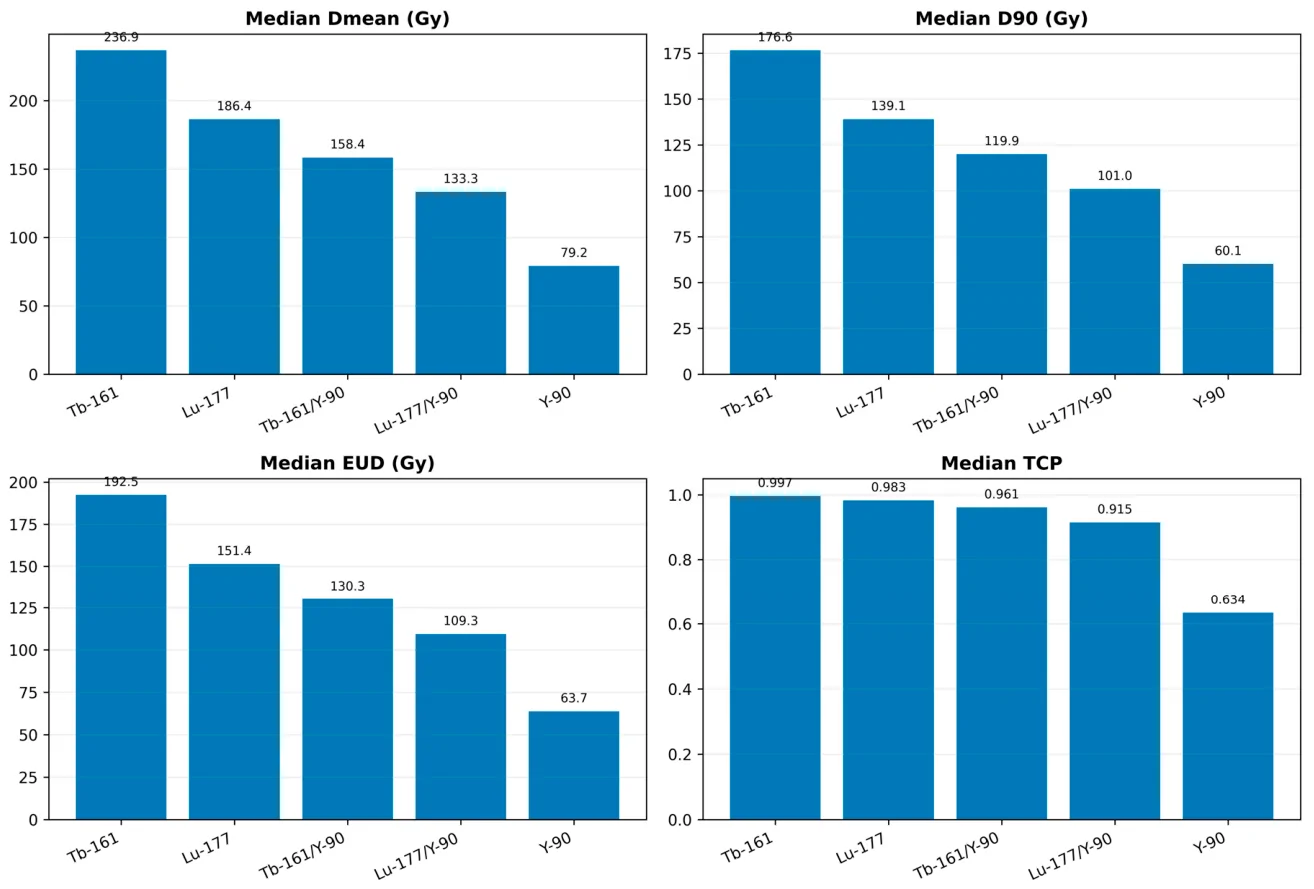
Comparison of lesion-level dosimetric and radiobiological outcomes across radionuclide treatment models. Median Dmean, D90, EUD, and TCP are shown for ^161^Tb, ^177^Lu, ^161^Tb/^90^Y, ^177^Lu/^90^Y, and ^90^Y models.

The median D90 was noticed to increase from 139.1 Gy for ^177^Lu to 176.6 Gy for ^161^Tb, representing a 27.0% increase in dose coverage. The corresponding median TCP increased from 0.983 to 0.997. In contrast, ^90^Y produced the lowest median D90 and TCP in the full lesion cohort, reflecting the fact that broad crossfire alone did not compensate for lower local dose concentration in the predominantly small-lesion dataset. Overall dose metrics and TCP values are presented in Table 1, while Figure 7 shows the patient-specific dose and TCP comparison across the treatment models.

**Figure 8:**
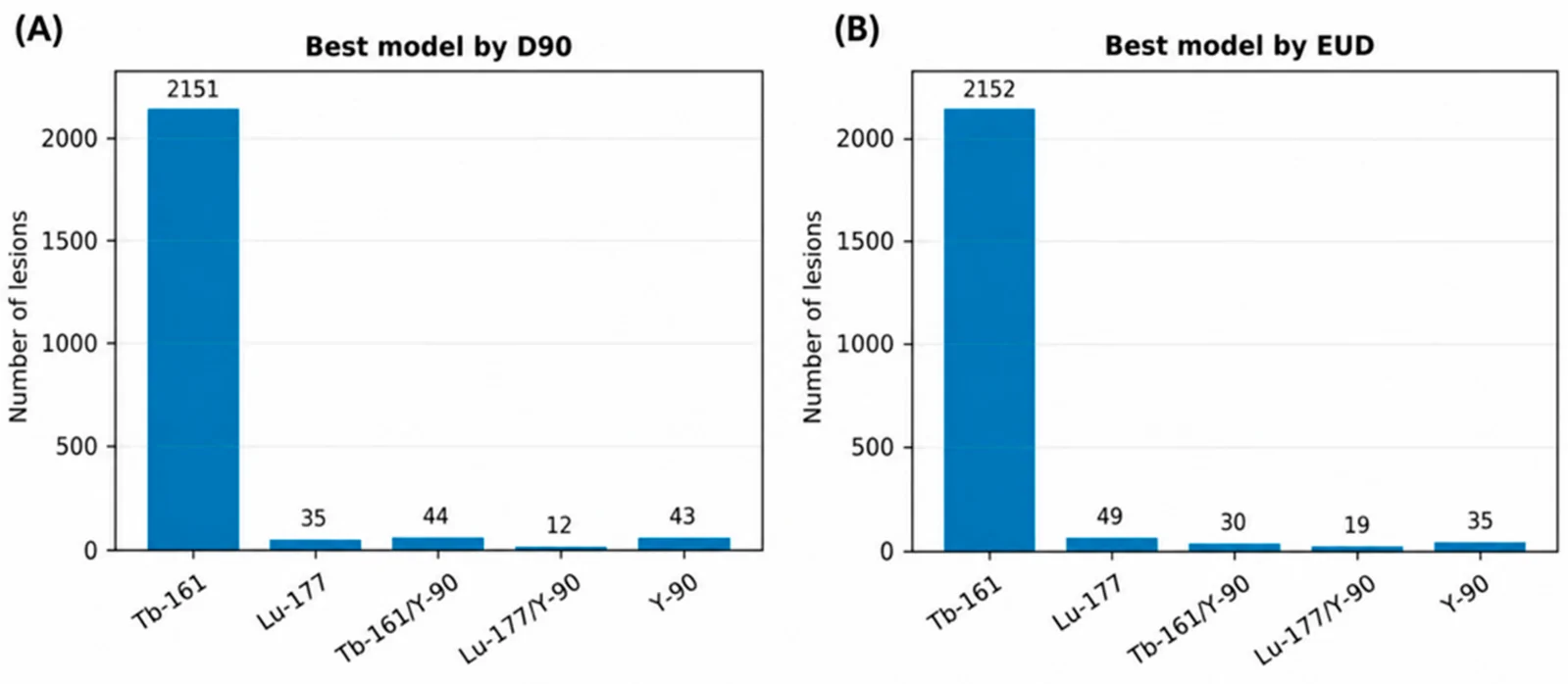
Lesion-level ranking of radionuclide treatment models. Number of lesions for which each model achieved the highest D90 (**A**) or EUD (**B**), demonstrating the predominance of ^161^Tb across both dosimetric endpoints.

Consideration at the lesion level highlighted the dominance of ^161^Tb (Figure 8). Using D90 as the dose deposition yardstick revealed ^161^Tb as the best-performing radioisotope for 2151 of 2285 lesions. The same pattern was also recorded with ^161^Tb for EUD when used as the endpoint. Nevertheless, a small but clinically important subset of lesions favoured a crossfire-bearing radioisotope, especially ^161^Tb/^90^Y or ^90^Y. This minority group is important because it identifies the biological niche in which a hybrid strategy may be clinically valuable, particularly for lesions in which deposited electron dose is not sufficient and extended crossfire improves coverage.

**Figure 9:**
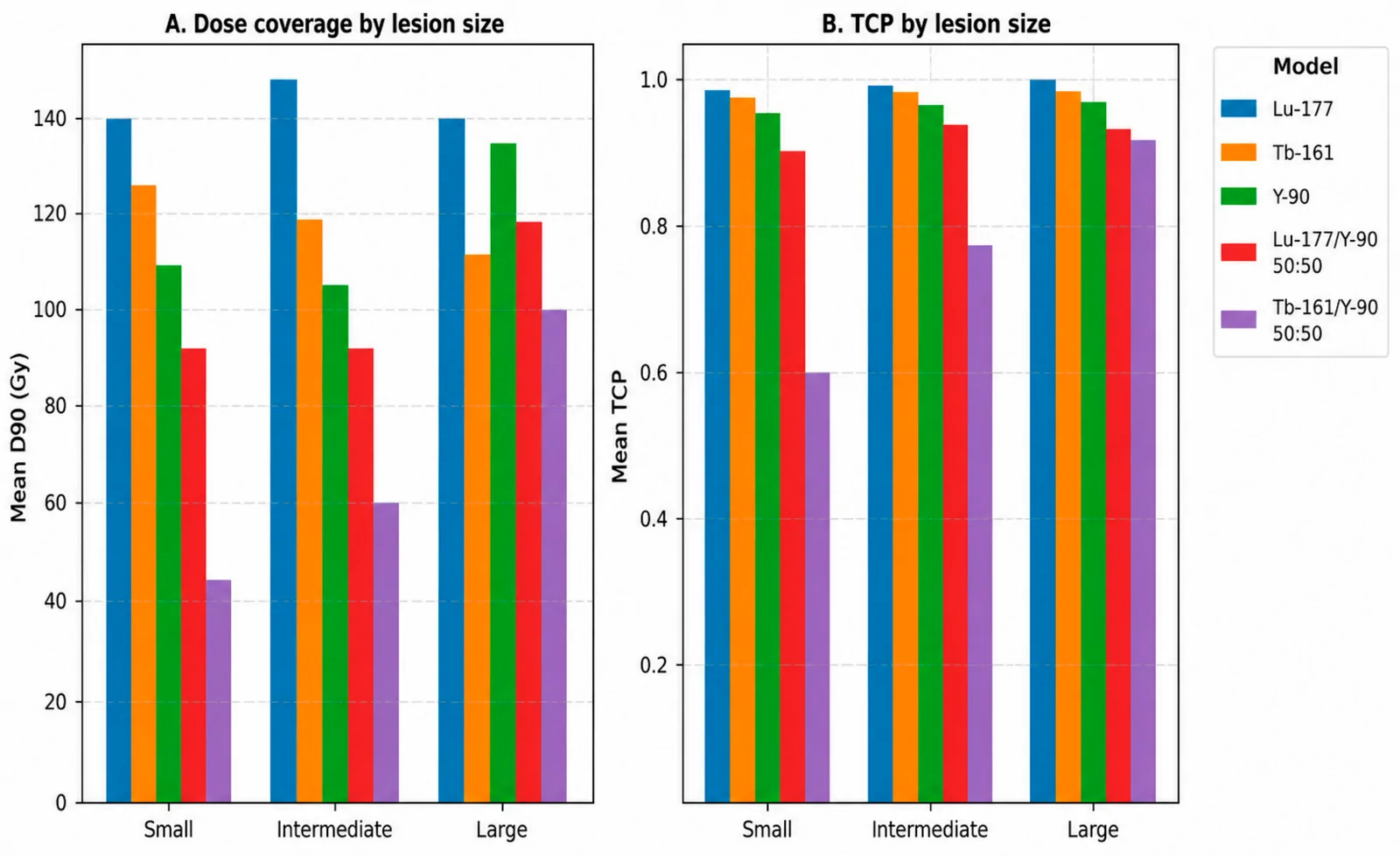
Size-stratified dosimetric and radiobiological performance across radionuclide treatment models. (**A**) Mean D90 and (**B**) mean TCP for small, intermediate and large lesions, illustrating lesion-size-dependent differences among single and hybrid radionuclide models.

Stratification of the results by size, as shown in Figure 9, presented a pattern for clinical consideration. It was observed that ^161^Tb was clearly dominant for small and intermediate lesions, producing the highest D90, EUD and TCP. In large lesions, the gap between ^161^Tb and crossfire-containing approaches narrowed. The median D90 in large lesions was 172.1 Gy for ^161^Tb, 168.6 Gy for ^161^Tb/^90^Y, 151.9 Gy for ^90^Y and 149.4 Gy for ^177^Lu/^90^Y. This convergence suggests that larger lesions may benefit from a degree of crossfire contribution, even when the overall cohort is best served by ^161^Tb.

**Figure 10:**
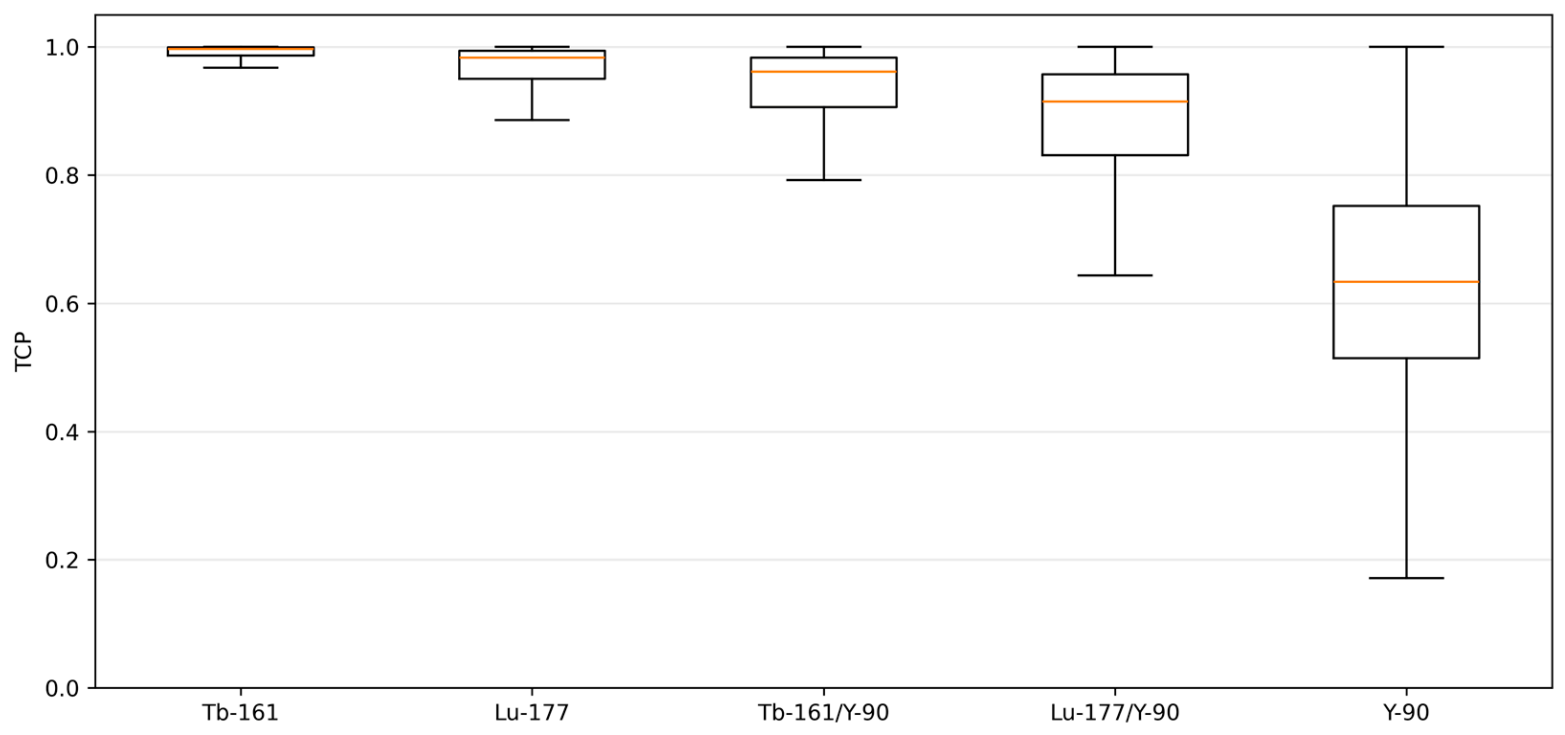
Lesion-level tumour control probability (TCP) distributions across radionuclide treatment models, showing higher and more consistent predicted TCP for ^161^Tb and greater variability for ^90^Y.

The TCP distribution presented in Figure 10 further emphasises the clinical demarcation between treatment models. It is revealed that ^161^Tb concentrated the greatest proportion of lesions near the complete predicted control, followed by ^177^Lu and ^161^Tb/^90^Y. The individual ^90^Y produced the broadest low-TCP distribution in the full dataset. This is consistent with lower local dose concentration in a lesion population dominated by small targets.

### 3.4. Sensitivity of Absorbed Dose to Temporal Kinetics

The kinetic sensitivity analysis revealed that the assumed effective clearance influenced the magnitude of the reconstructed absorbed dose while retaining the patient-specific spatial uptake distributions. For the short-retention condition (72 h biological half-life), effective half-lives were 2.067, 2.096 and 1.412 days for ^177^Lu, ^161^Tb and ^90^Y, respectively. Relative to the original effective half-life assumptions, this reduced the median absorbed dose by 40.9%, 40.1% and 29.4%, respectively. The intermediate retention (106 h) produced corresponding reductions of 24.2%, 22.8% and 16.8%, while the long retention (168 h) produced substantially smaller differences of 2.6%, 0.3% and 3.4%.

Across the investigated kinetic range, median D90 varied from 62.26 to 102.69 Gy for ^177^Lu, 81.60 to 135.82 Gy for ^161^Tb and 35.30 to 48.28 Gy for ^90^Y. Median EUD correspondingly ranged from 67.79 to 111.81 Gy, 88.94 to 148.03 Gy and 37.42 to 51.18 Gy, respectively. Despite the kinetic variation, ^161^Tb maintained the highest median D90 and EUD across all three retention conditions, followed by ^177^Lu and ^90^Y.

**Figure 11:**
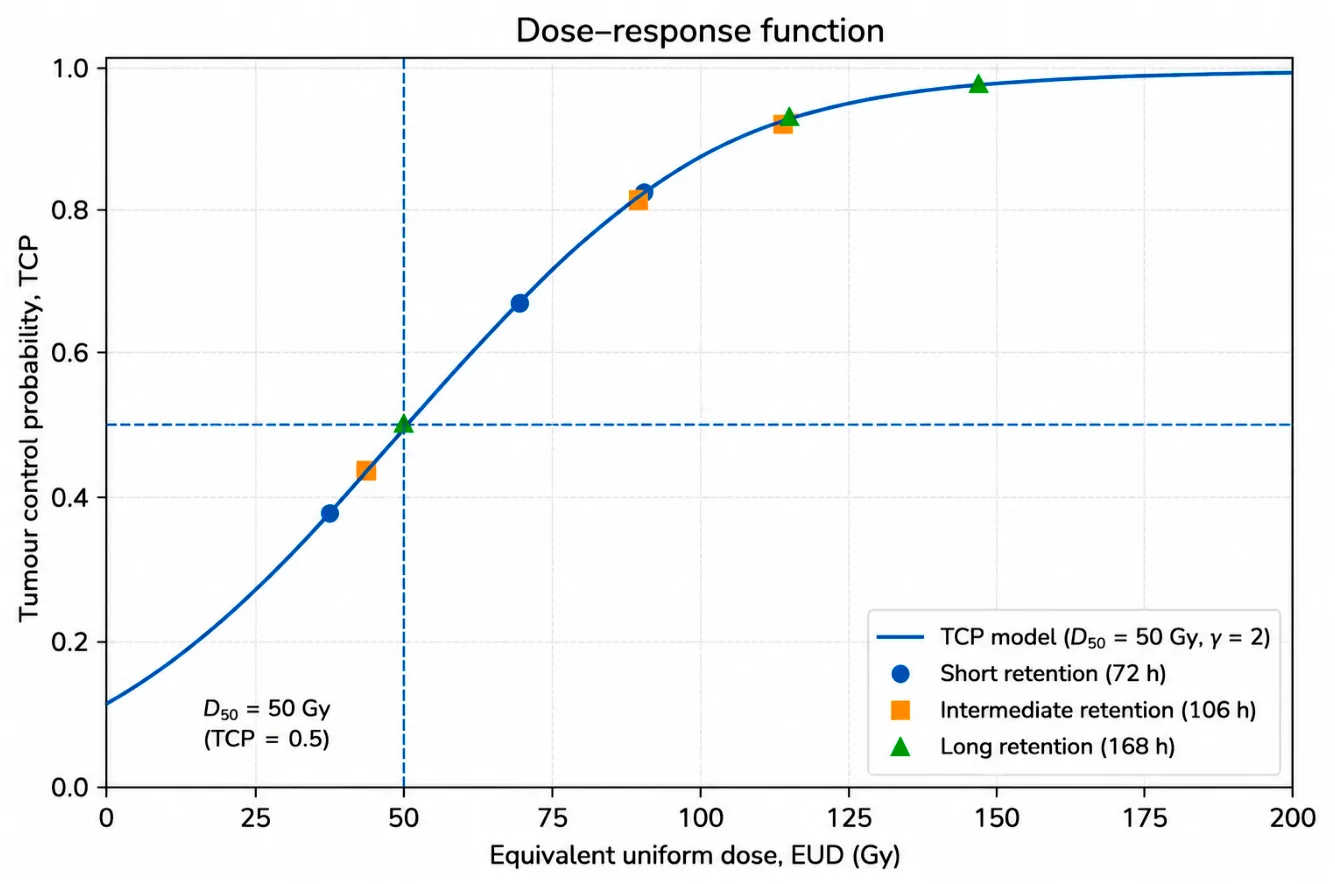
EUD–TCP dose–response relationship ($D_{50}$ = 50 Gy and γ=2), showing predicted responses for short (72 h)-, intermediate (106 h)-, and long (168 h)-retention cases.

The influence of the kinetic assumptions was more pronounced for the nonlinear TCP endpoint. The findings showed that median TCP ranged from 0.919 to 0.998 for ^177^Lu, 0.990 to approximately 1.000 for ^161^Tb, and 0.090 to 0.547 for ^90^Y across the short- to long-retention cases. The comparatively large variation in ^90^Y TCP reflects the position of its EUD values within the steep region of the adopted TCP dose–response function (Figure 11) rather than a proportionally equivalent change in absorbed dose.

These findings demonstrate that the absence of serial imaging introduces a quantifiable kinetic uncertainty into the absolute absorbed-dose estimates. Importantly, the radionuclide ranking was preserved across the investigated kinetic cases, favouring ^161^Tb consistently producing the highest median dose metrics, followed by ^177^Lu and ^90^Y.

### 3.5. Diagnostic Tracer Subgroup Comparison 

Analysis of the ^18^F and ^68^Ga subgroups, each comprising 10 patients, showed that, at the patient level, median SUV_mean_ (4.65 vs. 6.07; *p* = 0.678) and SUV_max_ (7.44 vs. 8.97; *p* = 0.473) did not differ significantly between the ^18^F and ^68^Ga groups, respectively, although median lesion volume was greater in the ^68^Ga compared to the ^18^F (2.26 against 1.08 mL; *p* = 0.017) group. The rank-biserial correlation coefficient was determined to be 0.64. No significant between-tracer differences were observed for patient-level Dmean, D90 or EUD for any of the three radionuclides (*p* > 0.3 for all). Importantly, the relative therapeutic radionuclide ranking was preserved within both diagnostic tracer groups. Median patient EUD followed ^161^Tb > ^177^Lu > ^90^Y for both ^18^F (129.8, 100.4 and 50.8 Gy, respectively) and ^68^Ga (99.4, 76.8 and 47.7 Gy, respectively). Thus, although the tracer cohorts differed in lesion-size distribution, the principal comparative dosimetric relationship between therapeutic radionuclides was maintained independently within each diagnostic tracer subgroup.

## 4. Limitations

Certain limitations of note when interpreting the findings from this study include the unavailability of serial quantitative measurements suitable for patient-specific time–activity curve fitting, thus limiting the use of available PSMA PET/CT data containing a single imaging time point. Consequently, patient-specific effective clearance and time-integrated activity could not be determined directly. The therapeutic activity of the radionuclides was therefore modelled using an assumed effective half-life. Robustness of the resulting dosimetry was examined through sensitivity analyses spanning different effective half-life values. These analyses demonstrated that the absolute absorbed-dose and radiobiological estimates were dependent on the assumed temporal kinetics, particularly under the shorter effective half-lives. Thus, the absolute doses and TCP values are presented as model-based estimates rather than definitive patient-specific therapeutic dosimetry.

In addition, the dose-point kernels were simulated in a homogeneous water medium, which is a controlled reference geometry for the comparison of radionuclide-specific energy deposition; this, however, does not explicitly account for patient-specific tissue composition, density heterogeneity, bone, lung, interfaces or other anatomical variations that may influence particle transport and absorbed-dose deposition. Although this approximation is expected to be more consequential for some emissions and anatomical environments than others, future implementation incorporating CT-derived heterogeneous material maps and patient-specific Monte Carlo transport is imperative to quantify the magnitude of this effect. Future validation should therefore incorporate matched serial therapeutic imaging, patient-specific heterogeneous Monte Carlo dosimetry, normal-organ dose constraints and clinical response data before application in clinical treatment prescription.

## 5. Conclusions

This study demonstrates that Monte Carlo-derived dose-point kernels integrated with patient-specific PSMA PET activity distributions provide a practical workplan for personalised targeted radionuclide therapy dosimetry. By reconstructing absorbed dose directly from lesion-specific uptake patterns rather than relying solely on administered activity, the proposed workplan preserves intratumoural heterogeneity and enables biologically meaningful evaluation of radionuclide performance at the individual lesion level.

Among the radionuclides investigated, ^161^Tb consistently achieved the highest absorbed-dose delivery, equivalent uniform dose and tumour control probability across the majority of the 2285 analysed lesions, confirming the dosimetric advantage conferred by its combination of β-particles with abundant conversion and Auger electrons. These emission characteristics produced excellent microscopic energy localisation while maintaining favourable tumour dose coverage, making ^161^Tb the preferred radionuclide for most small, intermediate and highly PSMA-avid lesions.

The study further demonstrates that radionuclide selection should be considered as a phenotype-dependent optimisation problem. Although ^90^Y produced a lower tumour dose in the predominantly small-lesion cohort, its long-range β-particle emissions generated substantially greater crossfire, making it increasingly competitive for bulky or highly heterogeneous tumours where activity distributions are spatially non-uniform. This finding highlights the importance of matching radionuclide physical characteristics to tumour phenotype rather than applying a single radionuclide to all patients.

The hybrid kernels were noted to produce controllable transitions between highly localised microscopic irradiation and extended macroscopic crossfire. Among the investigated combinations, the ^161^Tb/^90^Y (70:30) combination emerged as the most favourable balance between the microscopic localised and macroscopic heterogenous competing dosimetric objectives. Compared with the conventional 50:50 hybrid, the optimised kernel preserved substantially greater central dose localisation while maintaining clinically meaningful long-range dose coverage. This combination therefore provides a rational compromise for lesions requiring both effective irradiation of microscopic tumour deposits and adequate treatment of heterogeneous tumour regions.

Beyond targeted radionuclide therapy, the general modelling concept developed in this study may provide a platform for other spatially heterogeneous diseases in which therapeutic effectiveness depends on the distribution, penetration and complementary action of multiple agents. Potential applications include heterogeneous solid tumours treated with combination chemotherapy or targeted agents, antimicrobial treatment of spatially structured bacterial infections and biofilms, and sequential or combined antibiotic therapy in which agents with different penetration, pharmacokinetic and antimicrobial characteristics are employed. In such applications, the radionuclide-specific dose kernels would necessarily be replaced by appropriate agent-specific transport, diffusion, pharmacokinetic and pharmacodynamic response models. Thus, the broader value of the proposed framework lies in integrating patient-specific spatial information with complementary therapeutic characteristics to support the phenotype-informed optimisation of single, combined or sequential treatment strategies.

## Figures and Tables

**Table 1:** Overall absorbed-dose and radiobiological metrics across the treatment models.

Treatment Model	Dmean (Gy)	D90 (Gy)	D95 (Gy)	EUD (Gy)	TCP
^161^Tb	182.79 [149.97–214.24] 95% CI 145.81–239.95	136.28 [112.45–157.53] 95% CI 99.01–157.88	132.07 [106.92–152.14] 95% CI 95.82–154.87	148.53 [121.25–172.89] 95% CI 109.66–176.61	0.981 [0.945–0.993] 95% CI 0.916–0.994
^177^Lu	141.33 [115.94–165.75] 95% CI 112.81–185.64	105.42 [87.04–121.88] 95% CI 76.56–122.02	102.10 [82.64–117.65] 95% CI 74.02–119.84	114.78 [93.77–133.70] 95% CI 84.72–136.67	0.930 [0.852–0.966] 95% CI 0.800–0.970
^161^Tb/^90^Y	124.71 [101.32–148.93] 95% CI 110.70–178.08	94.29 [77.96–109.33] 95% CI 74.46–124.02	90.99 [74.22–105.45] 95% CI 71.16–118.52	102.45 [84.02–119.49] 95% CI 81.08–133.51	0.891 [0.796–0.942] 95% CI 0.776–0.966
^177^Lu/^90^Y	104.16 [84.34–124.57] 95% CI 92.73–150.24	78.83 [64.82–91.69] 95% CI 62.89–105.49	75.89 [62.29–88.24] 95% CI 59.78–99.74	85.38 [69.94–99.75] 95% CI 68.34–113.42	0.805 [0.689–0.880] 95% CI 0.676–0.927
^90^Y	65.85 [51.58–85.26] 95% CI 63.64–117.98	49.99 [40.25–61.32] 95% CI 45.76–82.88	47.53 [38.61–57.64] 95% CI 42.66–76.39	52.99 [42.79–64.69] 95% CI 46.88–85.30	0.530 [0.428–0.643] 95% CI 0.469–0.804

Values are medians [interquartile range]; 95% CIs are patient-cluster bootstrap confidence intervals for the cohort median. Note: Each treatment model comprised 2285 lesions from 20 patients. Patients were resampled with replacement while retaining all constituent lesions, thereby accounting for within-patient lesion clustering. Dmean, mean lesion absorbed dose; D90 and D95, minimum absorbed dose received by 90% and 95% of lesion volume, respectively; EUD, equivalent uniform dose; TCP, tumour control probability; CI, confidence interval.

## Data Availability

All analysis and generated data are found in the paper.

## References

[1] O’Donoghue J., Zanzonico P., Humm J., Kesner A. (2022). Dosimetry in radiopharmaceutical therapy. J. Nucl. Med..

[2] Mok G.S.P., Dewaraja Y.K. (2021). Recent advances in voxel-based targeted radionuclide therapy dosimetry. Quant. Imaging Med. Surg..

[3] Danieli R., Pistone D., Tranel J., Botta F., Uribe-Munoz C., Raspanti D., Salvat F., Wilderman S.J., Bardiès M., Amato E. (2024). Technical note: Impact of dose voxel kernel (DVK) values on dosimetry estimates in ^177^Lu and ^90^Y radiopharmaceutical therapy applications. Med. Phys..

[4] Graves S.A., Flynn R.T., Hyer D.E. (2019). Dose point kernels for 2174 radionuclides. Med. Phys..

[5] Kim K.M., Lee M.S., Suh M.S., Cheon G.J., Lee J.S. (2023). Voxel-Based Internal Dosimetry for ^177^Lu-Labeled Radiopharmaceutical Therapy Using Deep Residual Learning. Nucl. Med. Mol. Imaging.

[6] Ramonaheng K., Qebetu M., Ndlovu H., Swanepoel C., Smith L., Mdanda S., Mdlophane A., Sathekge M. (2024). Activity quantification and dosimetry in radiopharmaceutical therapy with reference to ^177^Lutetium. Front Nucl. Med..

[7] Huang M., Law H.K.W., Tam S.Y. (2025). Radiomics-Driven Tumor Prognosis Prediction Across Imaging Modalities: Advances in Sampling, Feature Selection, and Multi-Omics Integration. Cancers.

[8] Bolch W.E., Bouchet L.G., Robertson J.S., Wessels B.W., Siegel J.A., Howell R.W., Erdi A.K., Aydogan B., Costes S., Watson E.E. (1999). MIRD pamphlet No. 17: The dosimetry of nonuniform activity distributions—Radionuclide S values at the voxel level. J. Nucl. Med..

[9] Lanconelli N., Pacilio M., Lo Meo S., Botta F., Di Dia A., Aroche A.T., Pérez M.A., Cremonesi M. (2012). A free database of radionuclide voxel S values for the dosimetry of nonuniform activity distributions. Phys. Med. Biol..

[10] Wasserthal J., Breit H.C., Meyer M.T., Pradella M., Hinck D., Sauter A.W., Heye T., Boll D.T., Cyriac J., Yang S. (2023). TotalSegmentator: Robust Segmentation of 104 Anatomic Structures in CT Images. Radiol. Artif. Intell..

[11] Galloway M.M. (1975). Texture analysis using grey level run lengths. Comput. Graph. Image Process..

[12] Thibault G., Fertil B., Navarro C., Pereira S., Cau P., Lévy N., Sequeira J., Mari J. (2013). Shape and texture indexes application to cell nuclei classification. Int. J. Pattern Recognit. Artif. Intell..

[13] Zwanenburg A., Vallières M., Abdalah M.A., Aerts H.J.W.L., Andrearczyk V., Apte A., Ashrafinia S., Bakas S., Beukinga R.J., Boellaard R. (2020). The Image Biomarker Standardization Initiative: Standardized Quantitative Radiomics for High-Throughput Image-based Phenotyping. Radiology.

[14] Ceriani L., Verme P. (2012). The origins of the Gini index: Extracts from Variabilità e Mutabilità (1912) by Corrado Gini. J. Econ. Inequal..

[15] Lechthaler B., Pauly C., Mücklich F. (2020). Objective homogeneity quantification of a periodic surface using the Gini coefficient. Sci. Rep..

[16] Papadimitroulas P., Loudos G., Nikiforidis G.C., Kagadis G.C. (2012). A dose point kernel database using GATE Monte Carlo simulation toolkit for nuclear medicine applications: Comparison with other Monte Carlo codes. Med. Phys..

[17] Walrand S., Jamar F. (2021). Renal and Red Marrow Dosimetry in Peptide Receptor Radionuclide Therapy: 20 Years of History and Ahead. Int. J. Mol. Sci..

[18] Guerriero F., Ferrari M.E., Botta F., Fioroni F., Grassi E., Versari A., Sarnelli A., Pacilio M., Amato E., Strigari L. (2013). Kidney dosimetry in ^177^Lu and ^90^Y peptide receptor radionuclide therapy: Influence of image timing, time-activity integration method, and risk factors. BioMed Res. Int..

[19] Şenışık A.M., Şenışık M. (2026). Simulation-based dosimetric and radiobiological modeling of advanced radiotherapy delivery platforms for prostate cancer. Radiat. Phys. Chem..

[20] Lehenberger S., Barkhausen C., Cohrs S., Fischer E., Grünberg J., Hohn A., Köster U., Schibli R., Türler A., Zhernosekov K. (2011). The low-energy β^−^ and electron emitter ^161^Tb as an alternative to ^177^Lu for targeted radionuclide therapy. Nucl. Med. Biol..

[21] Alcocer-Ávila M.E., Ferreira A., Quinto M.A., Morgat C., Hindié E., Champion C. (2020). Radiation doses from ^161^Tb and ^177^Lu in single tumour cells and micrometastases. EJNMMI Phys..

[22] Haller S., Pellegrini G., Vermeulen C., van der Meulen N.P., Köster U., Bernhardt P., Schibli R., Müller C. (2016). Contribution of Auger/conversion electrons to renal side effects after radionuclide therapy: Preclinical comparison of ^161^Tb-folate and ^177^Lu-folate. EJNMMI Res..

[23] Velo A.F., Carter L.M., Humm J.L. (2025). Monte Carlo-based subcellular comparison of electron energy deposition by ^177^Lu and ^161^Tb: Implications for targeted radiopharmaceutical therapy. EJNMMI Phys..

[24] Dezarn W.A., Cessna J.T., DeWerd L.A., Feng W., Gates V.L., Halama J., Kennedy A.S., Nag S., Sarfaraz M., Sehgal V. (2011). Recommendations of the American Association of Physicists in Medicine on dosimetry, imaging, and quality assurance procedures for 90Y microsphere brachytherapy in the treatment of hepatic malignancies. Med. Phys..

[25] Ljungberg M., Celler A., Konijnenberg M.W., Eckerman K.F., Dewaraja Y.K., Sjögreen-Gleisner K., Bolch W.E., Brill A.B., Fahey F., SNMMI MIRD Committee (2016). MIRD Pamphlet No. 26: Joint EANM/MIRD guidelines for quantitative ^177^Lu SPECT applied for dosimetry of radiopharmaceutical therapy. J. Nucl. Med..

[26] Dieudonné A., Hobbs R.F., Bolch W.E., Sgouros G., Gardin I. (2010). Fine-resolution voxel S values for constructing absorbed dose distributions at variable voxel size. J. Nucl. Med..

[27] Hickson K.J., O’Keefe G.J. (2014). Effect of voxel size when calculating patient specific radionuclide dosimetry estimates using direct Monte Carlo simulation. Australas. Phys. Eng. Sci. Med..

[28] Buteau J.P., Kostos L., Alipour R., Jackson P., McInstosh L., Emmerson B., Haskali M.B., Xie J., Medhurst E., Ravi R. (2024). Clinical Trial Protocol for VIOLET: A Single-Center, Phase I/II Trial Evaluation of Radioligand Treatment in Patients with Metastatic Castration-Resistant Prostate Cancer with [^161^Tb]Tb-PSMA-I&T. J. Nucl. Med..

[29] Buteau J.P., Kostos L., Jackson P.A., Xie J., Haskali M.B., Alipour R., McIntosh L.E., Emmerson B., MacFarlane L., Martin C.A. (2025). First-in-human results of terbium-161 [^161^Tb]Tb-PSMA-I&T dual beta-Auger radioligand therapy in patients with metastatic castration-resistant prostate cancer (VIOLET): A single-centre, single-arm, phase ½ study. Lancet Oncol..

[30] Valente S., Borbinha J., Vaz P., Di Maria S. (2025). PENELOPE Dose assessment comparison for ^177^Lu and ^161^Tb using paediatric computational phantoms. Appl. Radiat. Isot..

